# Sensitization to nsLTP: A Retrospective Study in An Italian Pediatric Population over the Last Decade

**DOI:** 10.1155/2023/4053799

**Published:** 2023-05-16

**Authors:** Cristiana Indolfi, Giulio Dinardo, Angela Klain, Marcella Contieri, Giuseppina Rosaria Umano, Fabio Decimo, Salvatore Abbadessa, Carolina Vitulano, Giorgio Ciprandi, Michele Miraglia del Giudice

**Affiliations:** ^1^Department of Woman, Child and of General and Specialized Surgery, University of Campania Luigi Vanvitelli, Naples, Italy; ^2^Department of Precision Medicine, University of Campania “Luigi Vanvitelli”, Naples, Italy; ^3^Allergy Clinic, Private Clinic Villa Montallegro, Genoa, Italy

## Abstract

**Background:**

Food allergy is common in the Mediterranean, especially concerning lipid transfer proteins (LTPs) allergy. LTPs are widespread plant food allergens in fruits, vegetables, nuts, pollen, and latex. Also, LTPs are prevalent food allergens in the Mediterranean area. They can sensitize via the gastrointestinal tract and cause a wide range of conditions: from mild reactions, such as oral allergy syndrome, to severe reactions, such as anaphylaxis. LTP allergy in the adult population is well described in the literature, concerning both the prevalence and clinical characteristics. However, there is poor knowledge about its prevalence and clinical manifestation in children living in the Mediterranean.

**Materials and Methods:**

This study, including 800 children aged from 1 to 18 years, investigated the prevalence of 8 different molecules of nonspecific LTP over time in an Italian pediatric population visited over the last 11 years.

**Results:**

About 52% of the test population was sensitized to at least one LTP molecule. For all the LTPs analyzed, sensitization increased over time. In particular, using the years 2010 through 2020 as a comparison, the major increases were observed for the LTPs of the English walnut Jug r 3, the peanut Ara h 9, and the plane tree Pla a 3 (about 50%); the increase of the LTP of the Hazelnut Cor a 8 was about 36%, and that of the LTP of the artemisia Art v 3 was approximately 30%.

**Conclusions:**

The latest evidence in the literature indicates an increase in food allergy prevalence in the general population, including children. Therefore, the present survey represents an interesting perspective about the pediatric population of the Mediterranean area, exploring the trend of LTP allergy.

## 1. Introduction

Nonspecific lipid transfer proteins (nsLTPs) are ancient and highly conserved pan-allergenic molecules widespread in plant foods [1]. They are the primary food allergy (FA) in adults and adolescents in the Mediterranean Basin and the most important allergens that cause food-induced anaphylaxis in Italy [2]. This characteristic is due principally to their biochemical structure, such as high resistance to low pH, elevated temperature, and gastrointestinal proteolysis. In the review by Costa et al. [3], the physicochemical properties, including those of LTPs analyzed to identify how they influence the allergenic potency of plant allergens: the abundance of LTPs is related to an increased risk of allergic elicitation, while the loss of protein 3D structure does not affect their allergenicity; glycation, aggregation and high temperatures (100°C) do not affect their IgE-binding capacity, while the combination of pressure-heat and pressure-heat-enzymatic hydrolysis treatments is efficient in reducing the IgE-binding capacity of LTP. These implications are still the subject of clinical research. To date, they are classified into two subfamilies based on their molecular weight: nsLTP1 (9 kDa), which includes most of LTPs, capable of causing a specific IgE response, and nsLTP2 (7 kDa) [4]. LTPs are usually localized in the pericarp of the fruits, thanks to their defensive role against phytopathogenic bacteria and fungi. The manifestation and the clinical severity of the LTP-related symptoms vary according to the level of avidity of the IgE implicated. Many sensitized patients appear completely asymptomatic; others may manifest local symptoms as an oral allergy syndrome (OAS) or contact urticaria or, in more severe cases, significant manifestations like vomiting, asthma, abdominal pain, urticaria-angioedema, and systemic reactions up to anaphylactic shock [5]. The epidemiological and clinical aspects of FA are poorly studied in the pediatric population [1]. FA is an adverse immunologic response, which appears systematically after exposure to a certain food [6]. Symptoms of FA may vary, from common urticaria to anaphylaxis [7]. They can affect the gastrointestinal tract with vomiting and abdominal pain or the skin and mucosa with urticaria and edema. In severe reactions, also the cardiovascular system may be affected, with hypotension, tachycardia, up to cardiac arrest [8]. Ideally, any food can elicit an allergic reaction; the most common cause of FA in children are milk, egg, peanuts, tree nuts, shellfish, and fish [9, 10]. In the Mediterranean area, especially in Italy and Spain, rPru p 3, the first allergen to cause sensitivity in children is the nsLTP from peaches (*Prunus persica*). It may subsequently promote new sensitizations to many nsLTP-containing foods [11, 12]. Namely, rPru p 3 cross-reacts with other LTP molecules contained in many fruits belonging to the Rosaceae family, including apple, peach, apricot, and pollens, such as mugwort, olive, Parietaria, and plane [2, 13, 14]. This cross-reactivity is particularly evident for rPru av 3 (*Prunus avium*, cherry) and Mal d 3 (*Malus domestica*, apple), which have a structural homology of 88% and 80%, respectively. The structural homology of Pru p 3 with Jug r 3 (*Juglans regia*, walnut) appears lower (61%) as well as with Cor a 8 (*Corylus avellana*, hazelnut) (59%), Ara h 9 (*Arachis hypogaea*, peanut) (53%), Tri a 4 (*Triticum aestivum*, wheat) (45%), and it reduces more and more considering the LTP of pollens: Art v3 (*Artemisia vulgaris*, mugwort) (46%), Par j 1 (*Parietaria judaica*, pellitory wall) (29%), and Ole e 7 (*Olea europaea*, olive tree) (19%) [15]. Diagnosis of LTP allergy is based on clinical history, followed and partly supported by the skin prick test (SPT) with extracts or fresh food (prick-by-prick). However, Component resolved diagnosis (CRD) has improved the accuracy of diagnosing IgE-mediated FA [16], assessing sensitization to individual allergen molecules using purified native or recombinant allergens. Basophil activation test measures are helpful to differentiate between tolerant controls and patients with LTP allergies, although neither sensitivity nor reactivity can differentiate the severity of clinical symptoms [17]. The presence of allergen-specific IgE against LTPs could indicate a risk of allergic reactions; generally, the higher the level of IgE detected, the higher the probability of a clinically manifest allergic reaction [18, 19]. Recent data seem to suggest that there is a high probability of LTP-sensitized patients to progress over time to severe allergic reactions: in Betancor et al.'s [20] study, 13.2% of 38 plant-food LTP- sensitized patients experienced allergic reactions, and 31% of 113 plant-food-allergic patients sensitized to LTP reported reactions to new, previously tolerated plant foods. Moreover, several plant-food sensitizations may result from the cross-reaction of the LTPs from various plant foods and pollens, resulting in LTP syndrome; for example, Pru p 3 positivity can cause an allergy to any LTP-related food [21]. Furthermore, the same results for the same allergens may not provoke the same clinical manifestations due to differences in individual patient sensitivities [22, 23]. As there is poor knowledge concerning LTPs sensitization in pediatric populations in the Mediterranean area, the present study aimed to investigate the prevalence of LTPs in children in Campania, a region in southern Italy.

## 2. Material and Methods

### 2.1. Patients

This study included 800 consecutive pediatric patients who visited the pediatric allergology clinic at the University of Campania “Luigi Vanvitelli” from 2010 to 2020. All patients were between the ages of 1 and 18 years old and were being followed for atopic disorders such as allergic asthma, atopic dermatitis, and allergic rhinitis. They had a suspected diagnosis of FA proposed by their primary care pediatricians. The study retrospectively analyzed the serum-specific IgE for eight different nsLTPs using the microarray method (ImmunoCAP ISAC, ThermoFisher Scientific, Milan, Italy). First, the data concerning nsLTPs were extracted and then compared. The nsLTPs analyzed were Ara h 9 (peanut), Jug r 3 (walnut), Cor a 8 (hazelnut), Pru p 3 (peach), Tri a 14 (wheat), Art v 3 (mugwort), Ole e 7 (olive tree), and Pla a 3 (plane tree). Sensitization was diagnosed in the presence of a value greater than 0.35 ISU-E.

### 2.2. Endpoints

The primary objective of our study was to evaluate the trend of sensitizations to eight different nsLTPs in a pediatric population living in the Mediterranean area between 2010 and 2020. The secondary objective of our study was to compare sensitization year by year to assess the trend of each year and compare them.

### 2.3. Statistical Analysis

All continuous variables were evaluated for normality according to the Shapiro–Wilk test. Differences in not-normally distributed continuous variables were investigated using the Kruskal–Wallis test. Significance was set for *p*-values < 0.05. The data obtained about the nsLTPs analyzed during our study were compared using the chi-square test. All analyses were performed using Microsoft Excel for Microsoft 365, Microsoft Inc., Redmond, Washington, USA, and GraphPad Prism version 8.0.2 for Windows, GraphPad Software, San Diego, California, USA.

## 3. Results

**Figure 1:**
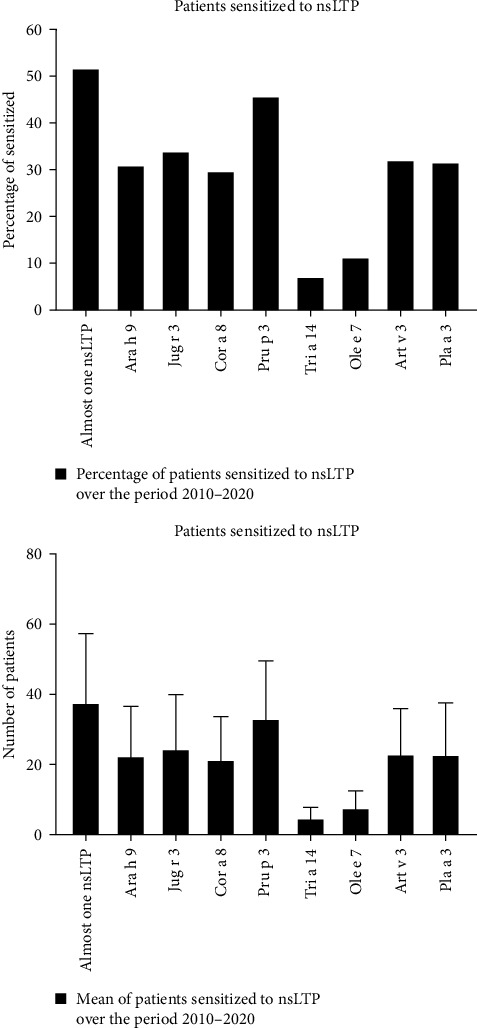
Sensitization to eight nsLTPs in the period 2010–2020.

Analysis of the results showed that 507 patients (63.4%) were male and 293 patients (36.6%) were female. The sensitization to peach Pru p 3 was the most common, affecting 46% of the population. Additionally, 34.2% of children were sensitized to Jug r 3, 32.4% to Art v 3, 31.9% to Pla a 3, 31.2% to Ara h 9, 30% to Cor a 8, 11.5% to Ole e 7, and 7.3% to Tri a 14. About 52% of the population in the study period was sensitized to at least one LTP (Figure 1).

**Figure 2:**
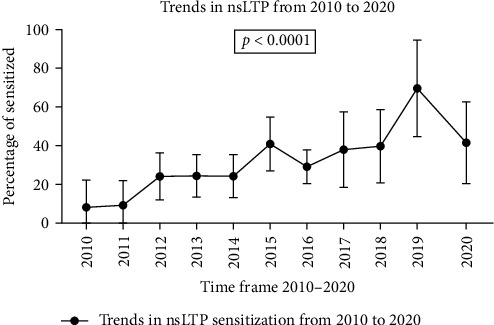
Trends in nsLTP sensitization from 2010 to 2020.

Over the past 10 years, the prevalence of LTP sensitization in the population has grown. According to the graph (Figure 2), the number of sensitized patients increased significantly for each nsLTP examined, peaking in 2019.

**Figure 3:**
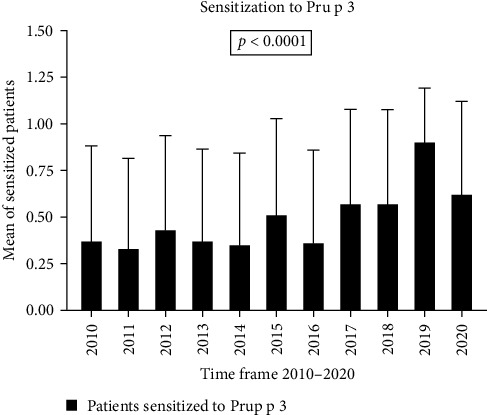
Trends in Pru p 3 sensitization over 2010–2020.

In particular, if we compare the years 2010 and 2020, the increase for the LTP of the English walnut Jug r 3 and the peanut Ara h 9 LTP was about 50%. The LTP of the Hazelnut Cor a 8 increased by about 36% when comparing 2010 to 2020, while during the same period, the LTP of Peach Pru p 3 had an increase of about 23% (Figure 3). The LTP of wheat Tri a 14 increased by about 6%, with a peak increase of about 20% in years such as 2015 and 2019. The LTP of the olive tree Ole e 7 shows an increase of about 11% when comparing 2010 to 2020. The LTP of Artemisia Art v 3 grew by around 30%, whereas the LTP of the plane tree Pla a 3 increased by approximately 50% during the same period (Table 1).

**Figure 4:**
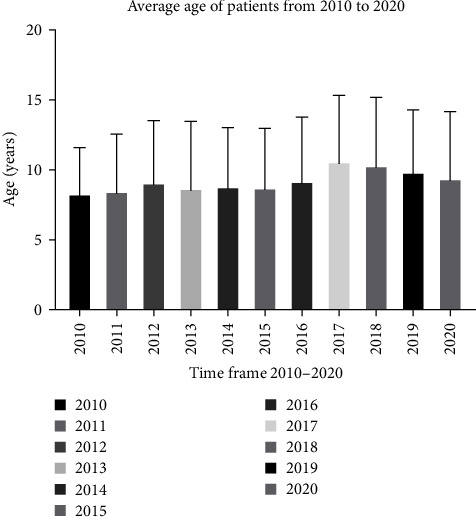
Average age of patients.

The data collected about the eight LTPs analyzed during our study show a *p*-value < 0.05 for each LTP analyzed from 2010 to 2020. The age difference of the patients between the single years studied by the multiple comparisons of the Kruskal–Wallis test obtained a nonsignificant value. In each of the years examined, the average age of the patients was consistently between 8 and 10 years (Figure 4).

## 4. Discussion

The objective of this study was to determine the prevalence of sensitization to LTP molecules among pediatric patients in Campania, southern Italy, from 2010 to 2020. The analysis of patient data revealed a frequency of approximately 52% for sensitization to LTP molecules over the entire period analyzed, with an increase observed in the second half of the decade for most of the molecules tested. To our knowledge, this is the first study to report on the prevalence of LTP sensitization in a pediatric population in Campania, southern Italy, over the past decade. Our findings are consistent with recent studies that have reported an increase sensitization and allergy in the last years [8, 24–27]. In the United States, the proportion of hospital admissions due to food anaphylaxis in children aged 0–18 years increased by more than two-fold between 2000 and 2009 [28]. Between 1998 and 2012, food anaphylaxis admission rates in the United Kingdom increased from 1.2 to 2.4/105, with 0–4 aged children showing the greatest rates [29]. The number of children who visited the American emergency departments for food-induced anaphylaxis increased by 214% (*p* < 0.001) from 2005 to 2014; infants and toddlers had the greatest rates [30]. Currently, the prevalence of FA in children is between 5% and 10% in Western countries and about 7% in China and Korea [25, 31]. The rise in FA and sensitization can be attributed to a number of factors, such as the lack of exposure to microbes necessary for building immune defenses in the early years of life, the polarization of the immune response toward a Th2 phenotype (hygiene hypothesis), vitamin D deficiency, indiscriminate use of antibiotics, pollution, delayed introduction of food allergens, and changes in the microbiota [24]. The principal limitation of our study is that we only discuss the prevalence of LTPs sensitizations in a selected pediatric population, not its relationship to clinical data and allergic symptoms. It is reasonable to assume a link between the increase in LTP sensitization and the rise in allergic reactions. However, it is crucial to differentiate between food sensitization and real FA, i.e., the occurrence of symptoms after ingestion of the sensitizing allergen. For example, in a recent article about 82 pediatric patients with allergic rhinitis due to Parietaria pollen allergy and sensitization to Pru p 3, the LTP of the peach, anaphylaxis after eating foods containing LTP was reported by about one-quarter of children, the other half reported FA or OAS; the remaining one-quarter were merely sensitized [32]. A Spanish study that examined a group of children with nut allergies discovered that clinical symptoms are not always present when a child becomes sensitized to a particular plant-food LTP: in fact, Pru p 3- and Jug r 3-IgEs were present in 69% and 63% of peach- and walnut-tolerant patients, respectively. Like this, 9.1% of hazelnut-tolerant people exhibited positive Cor a 8, compared to 36.8% for peanut (Ara h 9) and 26.2% for wheat (Tri a 14). Therefore, a definitive diagnosis of FA cannot be made based solely on IgE and SPT results without considering the patient's clinical history [33]. In addition, a study by Novembre et al. [34] found that the levels of specific IgE to Pru p 3 in Italian children with peach allergies do not help predict the severity of the allergic reactions. An allergy management strategy based on immunological understanding should be implemented for patients sensitized to LTP molecules. In other words, LTP-sensitized individuals (such as those who did not experience a clinical reaction) could consume any meal they could handle until overt symptoms started to show. This approach is clinically relevant as it helps to maintain both immunological and clinical tolerance. In addition, it is the base of the prevention of FA. To differentiate between tolerance and symptoms, a proper medical strategy should focus on increasing the patient's “engagement” with his actual clinical state [19, 35]. Dietary avoidance of essential nutrients, such as fruits and vegetables, may adversely affect a child's development, health, and quality of life for both the child and their parents. Because of these factors, food avoidance strategies should be based on clinical reactivity rather than only sensitization [6]. Due to the partial similarity of LTP derived from many foods and sensitization does not signify allergy, LTP-allergic people can consume all tolerated foods until the onset of overt symptoms. This strategy might avoid harmful and needless restrictive diets and possibly promote the growth of natural tolerance as a sort of physiological immunotherapy [19, 36]. The gold standard diagnostic test is still the oral food challenge in circumstances where diagnostic uncertainty persists.

## 5. Conclusion

LTP allergy is widely described in adults but is also an emerging issue in the pediatric population. The actual prevalence of this sensitization in children is not well known, and it still often remains underdiagnosed in these patients. By comparing literature documents with our experience, these data will have significant importance for subsequent epidemiological studies against some allergic diseases on our national territory and at the European level. LTP allergy can cause not only local allergic reactions such as urticaria and OAS but also more critical reactions such as nausea, vomiting, abdominal pain, angioedema, and systemic reactions such as anaphylactic shock. The analysis of these data allows us to evaluate the actual prevalence of sensitization toward the various LTP. This knowledge could be an important starting point for implementing innovative studies regarding the true prevalence of clinical reactions in the pediatric population and for implementing information and prevention strategies against possible allergic reactions in the pediatric population.

## Figures and Tables

**Table 1:** Year-to-year percentages of patients sensitized to nsLTP and mean age.

Years	2010	2011	2012	2013	2014	2015	2016	2017	2018	2019	2020
Mean age	8	8	9	8	8	8	9	10	10	9	9
Ara h 9 (%)	0	0	23	30	26	51	37	56	51	78	50
Jug r 3 (%)	0	1	23	36	32	55	37	54	56	89	55
Cor a 8 (%)	10	17	31	18	25	39	28	31	41	78	46
Pru p 3 (%)	40	33	43	37	36	51	37	58	58	91	63
Tri a 14 (%)	0	2	7	7	5	19	16	7	8	21	6
Ole e 7 (%)	0	0	9	12	11	22	18	14	14	41	11
Art v 3 (%)	15	21	34	26	24	39	25	37	40	79	46
Pla a 3 (%)	0	0	23	29	35	51	35	47	50	80	55

## Data Availability

Data are available from the corresponding author on reasonable request.
